# Artificial Intelligence-Aid Colonoscopy Vs. Conventional Colonoscopy for Polyp and Adenoma Detection: A Systematic Review of 7 Discordant Meta-Analyses

**DOI:** 10.3389/fmed.2021.775604

**Published:** 2022-01-13

**Authors:** Hui Pan, Mingyan Cai, Qi Liao, Yong Jiang, Yige Liu, Xiaolong Zhuang, Ying Yu

**Affiliations:** ^1^Department of Endoscopy, Shanghai Jiangong Hospital, Shanghai, China; ^2^Endoscopy Center, Zhongshan Hospital, Fudan University, Shanghai, China; ^3^Department of Gastroenterology, Shanghai Jiangong Hospital, Shanghai, China; ^4^Department of Surgery, Shanghai Jiangong Hospital, Shanghai, China

**Keywords:** colonoscopy, artificial intelligence, polyp detection, adenoma detection, discordant meta-analysis, Jadad algorithm

## Abstract

**Objectives:** Multiple meta-analyses which investigated the comparative efficacy and safety of artificial intelligence (AI)-aid colonoscopy (AIC) vs. conventional colonoscopy (CC) in the detection of polyp and adenoma have been published. However, a definitive conclusion has not yet been generated. This systematic review selected from discordant meta-analyses to draw a definitive conclusion about whether AIC is better than CC for the detection of polyp and adenoma.

**Methods:** We comprehensively searched potentially eligible literature in PubMed, Embase, Cochrane library, and China National Knowledgement Infrastructure (CNKI) databases from their inceptions until to April 2021. Assessment of Multiple Systematic Reviews (AMSTAR) instrument was used to assess the methodological quality. Preferred Reporting Items for Systematic Reviews and Meta-Analyses (PRISMA) checklist was used to assess the reporting quality. Two investigators independently used the Jadad decision algorithm to select high-quality meta-analyses which summarized the best available evidence.

**Results:** Seven meta-analyses met our selection criteria finally. AMSTAR score ranged from 8 to 10, and PRISMA score ranged from 23 to 26. According to the Jadad decision algorithm, two high-quality meta-analyses were selected. These two meta-analyses suggested that AIC was superior to CC for colonoscopy outcomes, especially for polyp detection rate (PDR) and adenoma detection rate (ADR).

**Conclusion:** Based on the best available evidence, we conclude that AIC should be preferentially selected for the route screening of colorectal lesions because it has potential value of increasing the polyp and adenoma detection. However, the continued improvement of AIC in differentiating the shape and pathology of colorectal lesions is needed.

## Introduction

According to the latest statistics from the World Health Organization (WHO), colorectal cancer (CRC) remains one of the leading reasons of cancer-related death (1). Colonoscopy has been regarded as the preferred modality for early detection and removal of premalignant lesions (2, 3). Evidence revealed that colonoscopy was not only associated with a reduced incidence of CRC, but was also associated with reduced risk of mortality of CRC (4). However, colonoscopy has been found to have high adenoma miss rate (5). As one of the most important measures of colonoscopy quality, studies estimated that for every 1% increase in the rate of adenoma detection, there is a 3% decrease in CRC-caused mortality and a 5% decrease in incidence of interval CRC (6). It is therefore imperative that adenoma miss rate during colonoscopy continue to be decreased (5, 7).

Currently, published studies have explored possible factors which may have an impact on performance of colonoscopy and demonstrated multiple factors which were associated with increased adenoma miss rate, including quality of bowel preparation before colonoscopy, blind spots of colon, recognition of colorectal lesions, and features of polyp and adenoma (8–10). Enormous efforts have been exerted to increase the detection of polyp and adenoma through the improvement of endoscopic techniques and quality of bowel preparation (9), however high miss rate for polyp and adenoma is still not significantly decreased. Moreover, diminutive colorectal polyp and adenoma is difficult to be detected visually, and even under the assistance of magnified and high-resolution endoscopy (11). Evidence suggested that repeated colonoscopy performed by a second endoscopist increased polyp detection rate (PDR) and adenoma detection rate (ADR) (12, 13). Therefore, more and more attention has been paid to the improvement of endoscopist's cognitive aspect and recognition for colorectal lesions (14–16).

With the development of artificial intelligence (AI), several real-time automatic polyp and adenoma detection systems based on convolutional neural network (CNN) have been developed and validated (17, 18), which are considered to have the ability of assisting endoscopist to efficiently and accurately detect colorectal lesions during colonoscopy (13). Several randomized controlled trials (RCTs) have investigated the value of these systems for assisting the detection of colorectal lesions in real world (9, 19–24). Meanwhile, on the basis of published RCTs, multiple meta-analyses have also been performed (25–31). Unfortunately, a definitive conclusion has not yet been generated from published meta-analyses. The comparative detection performance of AIC vs. conventional colonoscopy (CC) for polyps and adenomas remains under debate. We therefore performed this systematic review: (a) to systematically structure meta-analyses which compared AIC with CC; (b) to objectively select high-quality meta-analyses out from discordant meta-analyses; and (c) to determine the preferred colonoscopy modality of CRC screening using the currently available evidence.

## Materials and Methods

We developed the framework of the present systematic review according to the methodological framework recommended by the Cochrane handbook (32). We reported results according to the Preferred Reporting Items for Systematic Reviews and Meta-Analyses (PRISMA) guidelines (33, 34).

### Study Identification

We systematically searched potentially eligible studies in PubMed, Embase, Cochrane Library, and China National Knowledgement Infrastructure (CNKI) databases from their inceptions until to April 2021. We used the following search terms to construct search strategies: colonoscopy, artificial intelligence, convolutional neural network. Essential search strategy was modified to generate specific strategy according to the unique requirements of individual database. The search strategies of English databases are summarized in Supplementary Table 1. We also manually checked the references of all eligible studies in order to capture any eligible meta-analysis.

### Selection Criteria

According to the selection criteria developed in previous systematic review of discordant meta-analyses, we defined the following inclusion criteria: any meta-analysis which compared AIC with CC for the detection of polyps and adenomas during colonoscopy were considered to be eligible for our criteria. Meanwhile, we defined the following exclusion criteria: (a) narrative review; (b) meta-analysis incorporating non-RCTs into analysis; (c) systematic review without meta-analysis; and (d) meta-analysis which did not report clinical outcomes.

### Selection of Studies

Two reviewers independently selected studies. Any meta-analysis with full-text was selected if it met our selection criteria. Two reviewers independently extracted the following information from each eligible meta-analysis, including the name of the first author, publication year, journal of publication with the latest impact factor, level of evidence, time duration of literature search, selection criteria, database searched, design of primary studies, accumulated number of each eligible meta-analysis, software applied, additional analysis including heterogeneity analysis, sensitivity analysis, subgroup analysis, trail sequential analysis or the Grading of Recommendations Assessment, Development and Evaluation (GRADE). All outcomes of each meta-analysis were extracted.

### Assessment of Methodological Quality

Two independent reviewers assessed the methodological quality of each meta-analysis by using the Assessment of Multiple Systematic Reviews (AMSTAR) instrument (35). In AMSTAR instrument, the overall level of each meta-analysis is identified according to the matching level between actual information and 11 items. We also assess the quality of each meta-analysis by using the Oxford Evidence-based Medicine Levels of Evidence (36). Meanwhile, we used the Preferred Reporting Items for Systematic Reviews and Meta-Analyses (PRISMA) to measure the level of reporting quality.

### Application of the Jadad Decision Algorithm

We applied the Jadad decision algorithm to assist selecting high-quality meta-analyses from multiple discordant meta-analyses (37). Jadad decision algorithm is a useful tool for differentiating discordant meta-analyses. The Jadad decision algorithm was designed based on following four questions: (a) Do the meta-analyses ask the same question? (b) Do the meta-analyses include the same primary studies? (c) Do the meta-analyses incorporating the same primary trials achieve the same methodological quality? (c) Do the discordant meta-analyses incorporating different primary trials apply the same selection criteria? Two reviewers independently applied this decision algorithm, and then their results were cross-checked to ensure selecting meta-analysis with the highest quality of evidence to develop recommendations.

## Results

### Search Results

**Figure 1:**
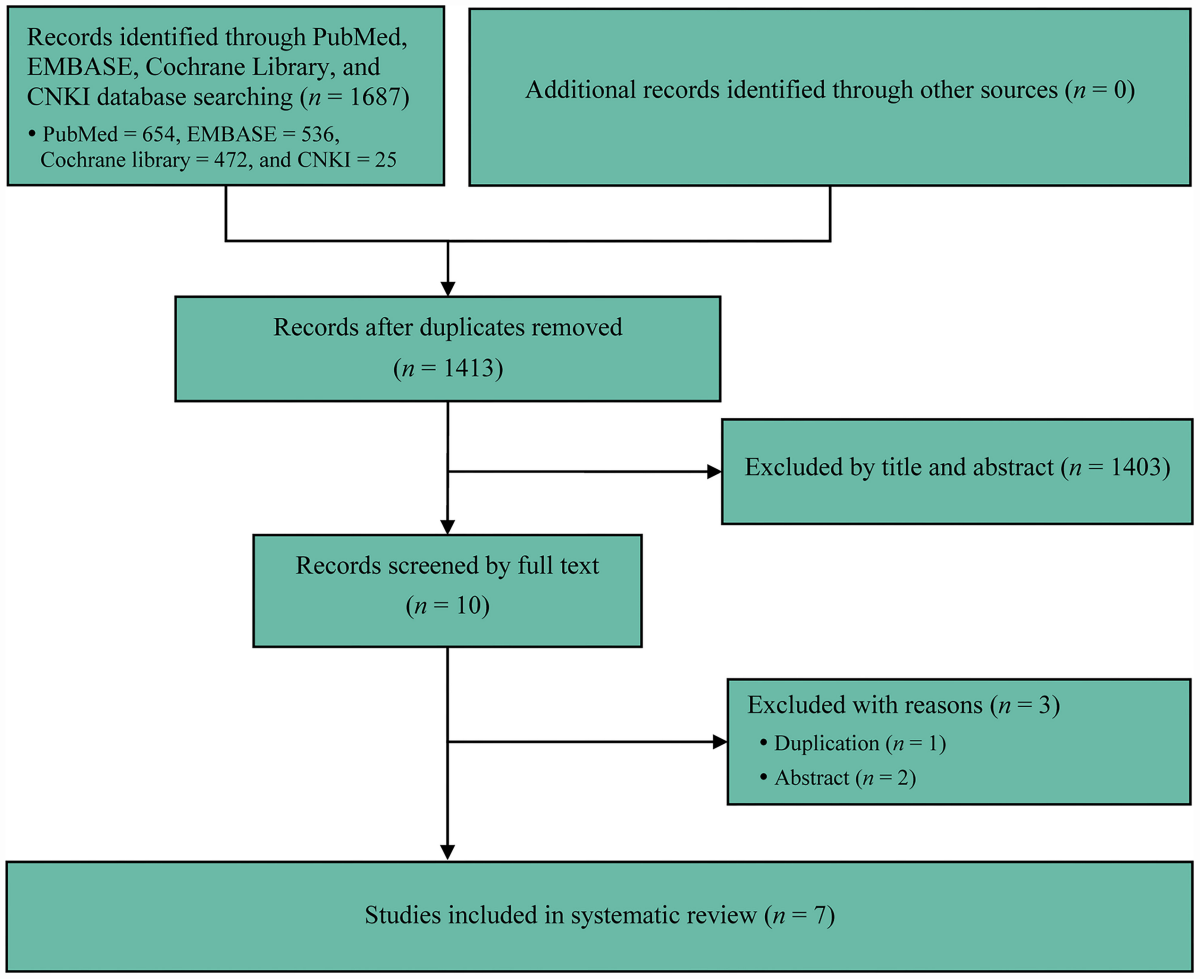
Flow diagram of identification and selection of meta-analysis.

We initially captured 1,687 records from target databases, and seven meta-analyses (2–31) were considered to meet our selection criteria eventually. We designed Figure 1 to delineate the process of retrieval and selection of eligible literature. Meanwhile, the reasons of excluding ineligible studies were also described in Figure 1. All included meta-analyses were published between 2020 and 2021 (Table 1). Five meta-analyses (26–31) were published in high-impact journals, which were defined to have an impact factor released by Web of Science (Table 1). The accumulated number of included primary RCTs in individual meta-analysis ranged from 3 to 7 (Tables 1, 2). The accumulated sample size of individual meta-analysis ranged from 2707 to 5427 (Table 1).

**Table 1:** General description of the characteristics of each meta-analysis.

**References**	**Journal abbreviation**	**Last time of search**	**No. of included RCTs**	**Accumulated sample size**	**Impact factor**
Ashat et al. (25)	*Endosc Int Open*	April 2020	6	5,142	n.a.
Aziz et al. (26)	*J Gastroenterol Hepatol*	January 2020	3	2,707	3.437
Barua et al. (27)	*Endoscopy*	February 2020	5	4,311	7.341
Deliwala et al. (28)	*Int J Colorectal Dis*	January 2021	6	4,996	2.108
Barua et al. (29)	*Gastrointest Endosc*	March 2020	5	4,354	6.890
Mohan et al. (30)	*EClinicalMedicine*	May 2020	6	4,962	n.a.
Zhang et al. (31)	*J Laparoendosc Adv Surg Tech*	July 2020	7	5,427	1.404

*n.a., not available*.

**Table 2:** Primary RCTs incorporated into each eligible meta-analysis.

**Primary RCTs**	**Included meta-analyses**						
	**Ashat et al. (25)**	**Aziz et al. (26)**	**Barua et al. (27)**	**Deliwala et al. (28)**	**Barua et al. (29)**	**Mohan et al. (30)**	**Zhang et al. (31)**
Wang et al. (38)	+	+	+	+	+	+	+
Gong et al. (19)	+		+	+			+
Wang et al. (9)	+		+	+	+	+	+
Liu et al. (21)	+	+	+	+	+	+	+
Su et al. (24)	+	+	+	+	+	+	+
Ashat et al. (23)	+			+	+	+	+
Wang, et al. (21)							+

### Search Methodology

All studies comprehensively searched databases. The number of databases which were searched in each meta-analysis ranged from 2 to 6. It is noted that Medline, EMBASE and the Cochrane Library were searched for capturing eligible primary studies among most meta-analyses (25–29). Unfortunately, other databases such as Scopus and Ovid platform were differentially selected by each meta-analysis. We summarized details of the search methodology in Table 3.

**Table 3:** Search methodology used by each meta-analysis.

**References**	**Restriction applied in meta-analyses**		**Database searched**					
	**Publication language**	**Publication status**	**PubMed**	**MEDLINE**	**EMBAS**	**Cochrane Library**	**WOS**	**Others**
Ashat et al. (25)	√	n.r.		+	+	+	+	+
Aziz et al. (26)	×	×	+	+	+	+	+	+
Barua et al. (27)	×	×		+	+	+		
Deliwala et al. (28)	n.r.	×		+	+	+	+	+
Barua et al. (29)	n.r.	×		+	+	+		
Mohan et al. (30)	√	×		+	+		+	+
Zhang et al. (31)	n.r.	×	+					+

*n.r., not reported; WOS, web of science*.

### Heterogeneity Assessment

Among all included meta-analyses, statistical heterogeneity was measured using the Cochrane Q and inconsistent index (*I*^2^ statistic). Total 4 softwares including RevMan, STATA, open meta-analyst (OMA), and comprehensive meta-analysis (CMA) were used for statistical analysis (Table 4). All meta-analyses (25–31) performed publication bias test and subgroup analysis, and three meta-analyses (27–29) performed sensitivity analysis (Table 4). Four meta-analyses (25–27, 29) used GRADE method to grade the level of evidence. Only one meta-analysis (28) registered protocol at public platform and used TSA method to test the robustness of pooled results (Table 4).

**Table 4:** Methodology for each meta-analysis.

**References**	**Design of included studies**	**Level of evidence**	**Software**	**GRADE**	**TSA**	**Additional analysis**			**Information of Register**
						**Subgroup analysis**	**Sensitivity analysis**	**Publication** **bias**	
Ashat et al. (25)	RCT	Level I	RevMan	+		+		+	
Aziz et al. (26)	RCT	Level I	OMA, CMA	+		+		+	
Barua et al. (27)	RCT	Level I	STATA	+		+	+	+	
Deliwala et al. (28)	RCT	Level I	CMA		+	+	+	+	+
Barua et al. (29)	RCT	Level I	STATA, RevMan	+		+	+	+	
Mohan et al. (30)	RCT	Level I	RevMan, CMA			+		+	
Zhang et al. (31)	RCT	Level I	RevMan			+		+	

*OMA, open meta analyst; CMA, comprehensive meta-analysis*.

### Study Quality and Validity

All eligible meta-analyses were performed based on RCTs. The AMSTAR score ranged from 8 to 10 points, which is documented in Table 5. According to the Oxford Levels of Evidence, all meta-analyses (25–31) were assessed as Level I evidence (Table 1). According to the PRISMA criteria, the score of individual meta-analysis was between 23 and 26, indicating a high-level reporting quality (Table 6).

**Table 5:** AMSTAR criteria for each meta-analysis.

**Items**	**Ashat et al. (25)**	**Aziz et al. (26)**	**Barua et al. (27)**	**Deliwala et al. (28)**	**Barua et al. (29)**	**Mohan et al. (30)**	**Zhang et al. (31)**
Was an a prior design provided?	0	0	0	1	0	0	0
Was there duplicate study selection and data extraction?	1	1	1	1	1	1	1
Was a comprehensive literature search performed?	1	1	1	1	1	1	1
Was the status of publication (i.e., Gray literature) used as an inclusion criterion?	0	1	1	1	1	1	1
Was a list of studies (included and excluded) provided?	0	0	0	0	0	0	0
Were the characteristics of the included studies provided?	1	1	1	1	1	1	1
Was the scientific quality of the included studies assessed and documented?	1	1	1	1	1	1	1
Was the scientific quality of the included studies used appropriately in formulating conclusions?	1	1	1	1	1	1	1
Were the methods used to combine the findings of studies appropriate?	1	1	1	1	1	1	1
Was the likelihood of publication bias assessed?	1	1	1	1	1	1	1
Was the conflict of interest stated?	1	1	1	1	1	1	1
Total score	8	9	9	10	9	9	9

**Table 6:** PRISMA criteria for each included meta-analysis.

**Reporting items**	**Ashat et al. (25)**	**Aziz et al. (26)**	**Barua et al. (27)**	**Deliwala et al. (28)**	**Barua et al. (29)**	**Mohan et al. (30)**	**Zhang et al. (31)**
Title	1	1	1	1	1	1	1
Abstract	1	1	1	1	1	1	1
Rationale	1	1	1	1	1	1	1
Objectives	1	1	1	1	1	1	1
Eligibility criteria	1	1	1	1	1	1	1
Information sources	1	1	1	1	1	1	1
Search strategy	1	1	1	1	1	1	1
Selection process	1	1	1	1	1	1	1
Data collection process	1	1	1	1	1	1	1
Data items	1	1	1	1	1	1	1
Study risk of bias assessment	1	1	1	1	1	1	1
Effect measures	1	1	1	1	1	1	1
Synthesis methods	1	1	1	1	1	1	1
Reporting bias assessment	1	1	1	1	1	1	1
Certainty assessment	1	1	1	0	1	0	0
Study selection	1	1	1	1	1	1	1
Study characteristics	1	1	1	1	1	1	1
Risk of bias in studies	1	1	1	1	1	1	1
Results of individual studies	1	1	1	1	1	1	1
Synthesis of results	1	1	1	1	1	1	1
Reporting biases	1	1	1	1	1	1	1
Certainty of evidence	1	1	1	0	1	0	0
Discussion	1	1	1	1	1	1	1
Protocol and registration	0	0	0	1	0	0	0
Support	0	1	1	0	1	1	1
Competing interests	1	1	1	1	1	1	1
Availability of data, code and other materials	0	0	1	0	0	1	0
Total score	24	25	26	23	25	24	23

### Results of the Jadad Decision Algorithm

**Figure 2:**
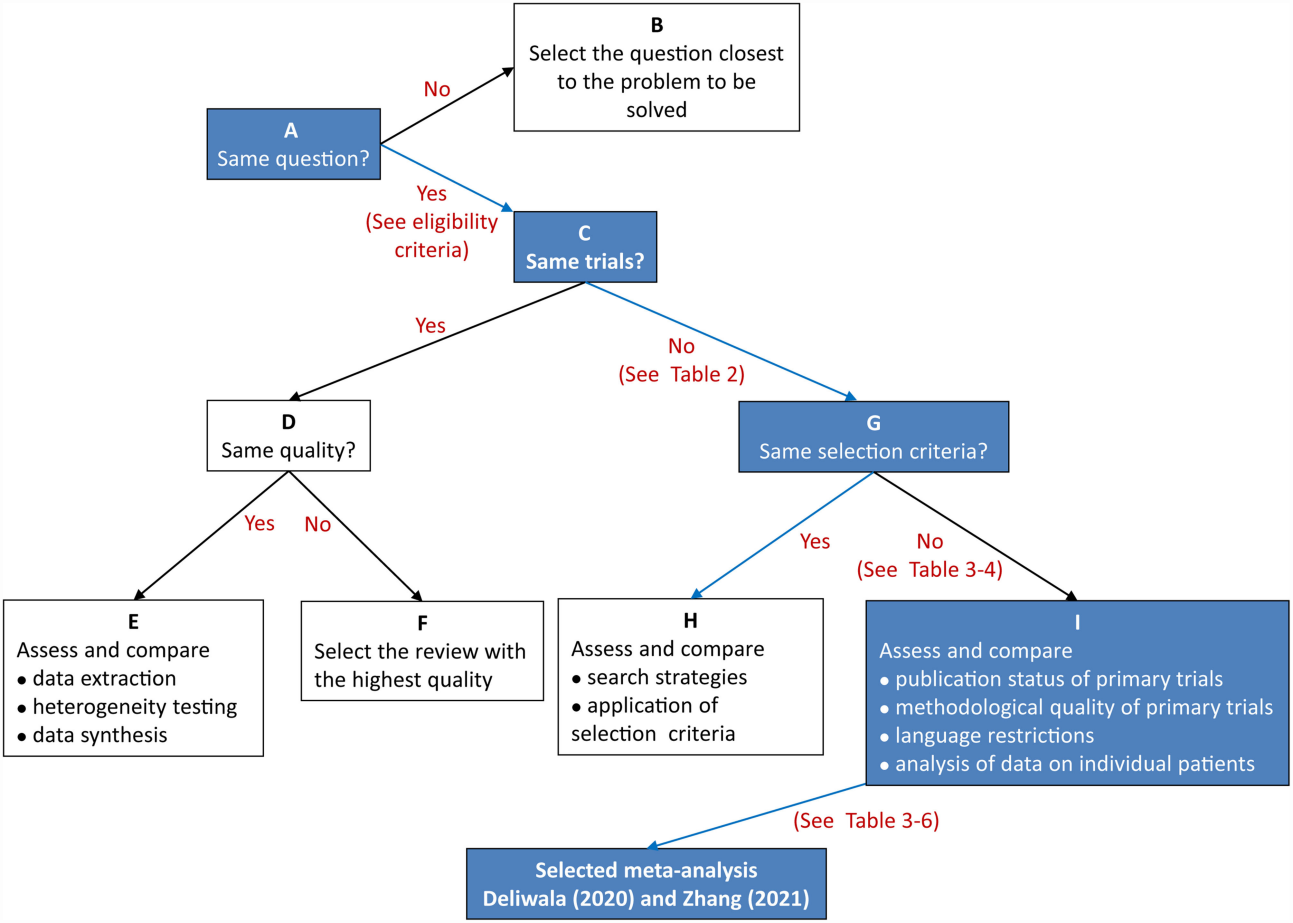
Flow diagram of Jadad decision algorithm.

Two reviewers independently performed the same flow path according to the Jadad decision algorithm. Because (a) all included meta-analyses investigated the same question (comparing AIC with CC for polyp and adenoma detection), (b) the included meta-analyses did not include the same RCTs, and (c) they did not design the same selection criteria, we selected the meta-analysis with the highest quality based on the following factors: publication status of the primary studies, methodology of the primary studies, language restrictions and the analysis of data on individual patients (37). Two eligible meta-analyses imposed language restriction, however all eligible meta-analyses did not restrict the publication status of primary studies. Therefore, we firstly excluded two meta-analyses performed by Ashat (25) and Mohan (30), respectively. Another three meta-analyses (26, 27, 29) were also excluded because of they did not completely include primary studies. Finally, two reviewers selected the remaining meta-analyses by Deliwala (28, 31), respectively, as the meta-analyses providing the best available evidence (Figure 2).

**Figure 3:**
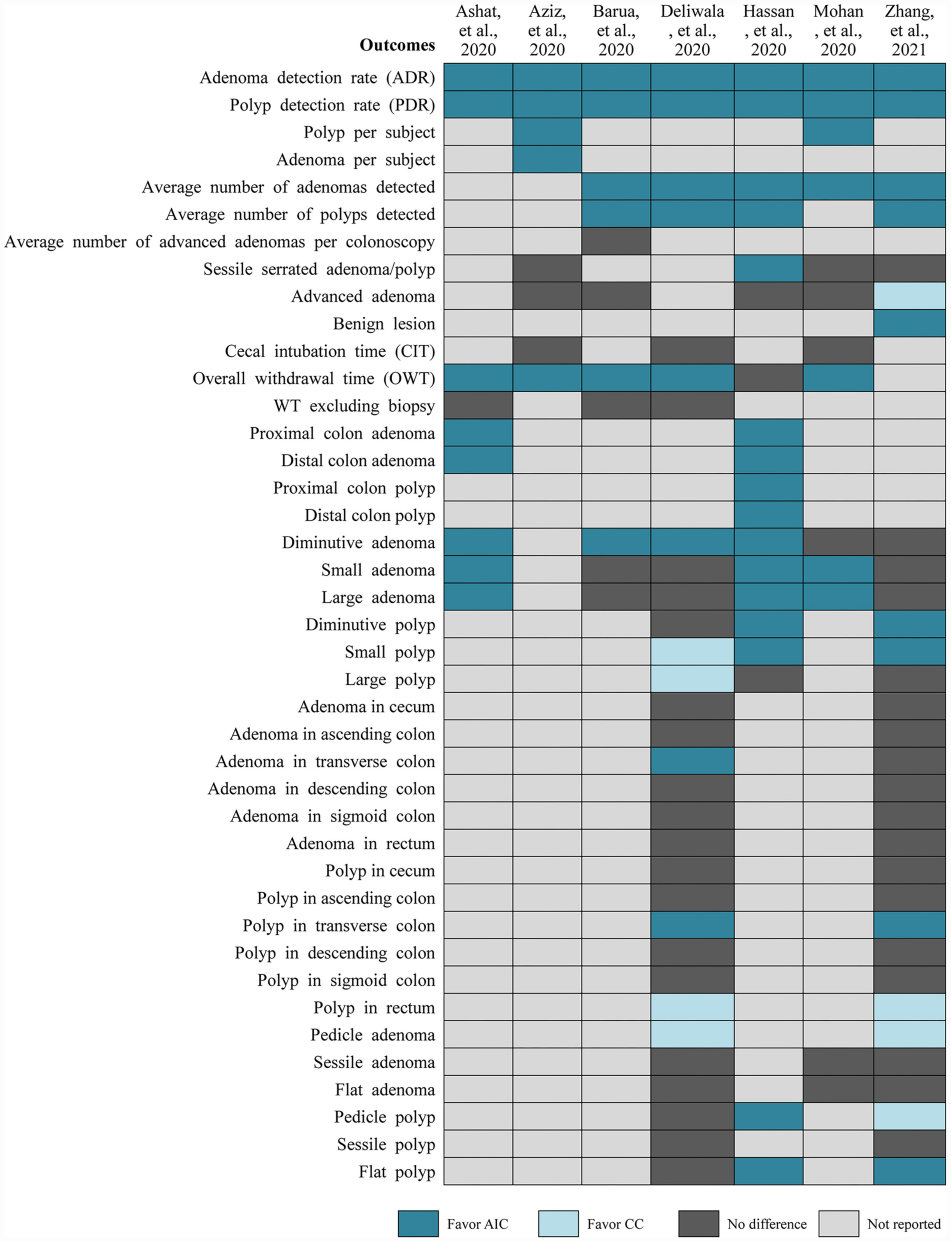
Results of each included meta-analysis.

Among the selected meta-analyses (Figure 3), PDR and ADR were all significantly increased in the AIC group compared to CC group, which was all confirmed by the results from TSA which was reported in the study performed by Deliwala (28). Meanwhile, these two meta-analyses consistently suggested that AIC was associated with increased number of polyps and adenomas detected per colonoscopy. Zhang and colleagues found that AIC benefits to detect benign lesions compared to CC, however CC may be better than AIC in detecting advanced adenoma (31). Meta-analysis by Deliwala indicated that AIC benefits to achieve target withdrawal time (6 min) compared to CC (28). For the detection of diminutive polyp and adenoma and small to large polyps, two meta-analyses reported conflicting results. Two meta-analyses suggested that AIC increased the detection of polyp in the transverse colon, however detected more polyps in rectum and pedicle adenomas. For detection of adenoma in transverse colon and pedicle and flat polyps, two meta-analyses reported conflicting results.

## Discussion

Currently, several RCTs (9, 19–24, 38) which investigated the comparative efficacy and safety of AIC vs. CC for the detection of polyps and adenomas during colonoscopy have been reported. Unfortunately, conflicting results were reported in these primary studies. Meanwhile, multiple meta-analyses (25–31) based on published RCTs have also been performed to systematically evaluate the role of AI-aid automatic detection system in assisting the detection of polyp and adenoma. Published meta-analyses comprehensively searched potentially eligible studies in main databases such as PubMed, EMBASE and Cochrane library, however they enrolled different RCTs and obtained conflicting conclusions, which has confused decision makers to appropriately make recommendations.

Fortunately, those possible sources of causing discordant results among meta-analyses have been deeply interpreted and clearly reported by Jadad and colleagues (37). Based on those possible sources, Jadad and colleagues developed a decision algorithm to assist researchers and practitioners selecting high-quality evidence from multiple discordant meta-analyses. To date, Jadad decision algorithm has been extensively applied to select the currently best available evidence from discordant meta-analyses. In the present systematic review, we eventually identified 7 meta-analyses (25–31) to meet our selection criteria. Unfortunately, among these 7 meta-analyses, three studies (26, 27, 29) were excluded because of they missed some eligible primary studies, which greatly impair the reliability of pooled results (32) although they used GRADE method to grade the level of evidence. As a gold method of pooling the currently best evidence, meta-analysis has been deeply developed and extensively used in route practice. However, it is critically important to capture all potentially eligible studies without restriction of language and publication status to generate robust and reliable pooled estimates (32). Unfortunately, another two meta-analyses (25, 30) were subsequently excluded because of Ashat and Mohan imposed language restriction in their meta-analyses. Although meta-analysis by Deliwala (28) missed one eligible primary study, TSA method was applied to examine the robustness of pooled results. We therefore selected it as candidate of providing currently best evidence. Meanwhile, meta-analysis by Zhang (31) included the most number of eligible primary studies and did not have significant drawbacks in terms of methodology, and thus it was also selected to provide currently best evidence for decision-making.

As one of the most common quality metrics of colonoscopy (6), PDR and ADR were reported to have significant improvement in AIC group compared to CC group in our selected two high-quality meta-analyses (28, 31). Meanwhile, Deliwala and colleagues applied TSA method to further confirm the reliability and robustness of pooled results of PDR and ADR (28). It must be noted that missed colorectal lesions especially sessile polyp and adenoma have been found to be associated with increased risk of interval CRC (7, 24), and thus it is critically important to greatly increase the detection of possible colorectal lesions during colonoscopy. Actually, several methods have been used to increase ADR in clinical practice, and the relative efficacy of these methods have also been comprehensively investigated in two published network meta-analyses currently (39, 40). However, in the present systematic review of discordant meta-analyses, we aimed to further determine the performance of AIC in the improvement of ADR compared with CC. Among selected meta-analyses, average numbers of polyps and adenomas detected per colonoscopy were all significantly increased in the AIC group related to the CC group. Blind spot of the colon is also important factor of reducing detection of colorectal lesions (8, 9). Automatic real-time polyp and adenoma detection system based on deep CNN has been found to have ability of offsetting powerless of the human eye for blind spot (15, 17). Selected meta-analyses also suggested that AIC increase the detection of polyps and adenoma in the transverse colon. Moreover, the size, shape and pathology of polyp and adenoma were all proved to be closely related to the canceration (29, 30). However, selected meta-analyses did not report consistent results for these outcomes (28, 31). So, these two meta-analyses concluded that AIC could significantly increase the PDR and ADR, especially for diminutive polyps and adenomas, however recognition for shape and pathology through AIC should be further improved.

Our current study has some strength: First, it is the first systematic review to differentiate multiple discordant meta-analyses investigating the comparative efficacy and safety of AIC vs. CC for the detection of polyp and adenoma. Second, our systematic review selects the currently best high-quality evidence from multiple discordant meta-analyses for determining a preferred colonoscopy modality during CRC screening. Our study is a systematic review of discordant meta-analyses, which is utilized to appraise the methodological quality and quality of reporting of meta-analyses. It is distinct from traditional systematic reviews that analyze primary studies. In this systematic review of discordant meta-analyses, we selected the best one from the currently published meta-analyses and provided high-level evidence to decision makers. Therefore, the results of our present study are more strength than that from previous meta-analyses or RCTs.

Certainly, we must acknowledge that our systematic review has also several limitations. First and foremost, prospective registration of protocol is the prerequisite of objectively and transparently conducting a systematic review and subsequently reporting results. However, we did not register our protocol on a public platform, which may negatively affect the confidence of our findings. Second, although we selected a meta-analysis incorporating TSA method to provide currently best evidence, all meta-analyses incorporating GRADE method was excluded, and thus we could not determine the confidence for each outcome. Third, we selected two meta-analyses to provide the best available evidence for decision makers finally, however one with the maximum number of primary studies did not register corresponding protocol on public platform (PROSPERO, an international prospective register of systematic reviews). As mentioned previously, prospective registration could guarantee the objectivity and transparent transparency of a meta-analysis, and thus results from that one without registration should be cautiously interpreted.

## Conclusions

In conclusion, this systematic review of discordant meta-analyses concludes that AI-based polyp detection systems during colonoscopy increase the detection of polyps and adenomas, especially for diminutive and small, non-advanced polyps and adenomas. However, the potential value of AIC in recognizing the shape and pathology of colorectal lesions should be further improved.

## Data Availability Statement

The original contributions presented in the study are included in the article/Supplementary Material, further inquiries can be directed to the corresponding author/s.

## Author Contributions

HP and MC conceived the study and revised the initial manuscript. QL, YJ, and YL captured and selected citations. XZ and YY designed the data extraction table and extracted data. HP, MC, QL, YJ, and YL performed statistical analyses and prepared for the manuscript draft. All authors approved the final version of the manuscript.

## Funding

This study was support by Shanghai Youth Medical Talents-Specialist Program (2019[72]), Zhongshan Hospital Youth Medical Talents (2019ZSYQ11) and National Key Research and Development Plan of China (No.2017YFC1308802).

## Conflict of Interest

The authors declare that the research was conducted in the absence of any commercial or financial relationships that could be construed as a potential conflict of interest.

## Publisher's Note

All claims expressed in this article are solely those of the authors and do not necessarily represent those of their affiliated organizations, or those of the publisher, the editors and the reviewers. Any product that may be evaluated in this article, or claim that may be made by its manufacturer, is not guaranteed or endorsed by the publisher.
