# Supplementary material for: Artificial Intelligence-Aid Colonoscopy Vs. Conventional Colonoscopy for Polyp and Adenoma Detection: A Systematic Review of 7 Discordant Meta-Analyses
Source: Front Med (Lausanne). 2022 Jan 13;8:775604. doi: 10.3389/fmed.2021.775604 (PMC8792899; doi:10.3389/fmed.2021.775604)
Supplement: Supplementary file 1 [file Table_1.DOCX]

**Supplementary Table 1.** Search strategies for target databases

| **Pubmed search strategy** | |
| --- | --- |
| No. | Query |
| 8 | #3 AND #6 AND #7 |
| 7 | ((((((("Randomized Controlled Trial" [Publication Type]) OR "Randomized Controlled Trials as Topic"[Mesh]) OR "Random Allocation"[Mesh]) OR "Double-Blind Method"[Mesh]) OR "Single-Blind Method"[Mesh]) OR "Placebos"[Mesh]) OR ((random*[Text Word]) OR (placebo[Title/Abstract]))) OR (((((singl*[Text Word]) OR (doubl*[Text Word])) OR (trebl*[Text Word])) OR (tripl*[Text Word])) AND (((mask*[Text Word]) OR (blind*[Text Word])) OR (dumm*[Text Word]))) |
| 6 | #4 OR #5 |
| 5 | (((((Colonoscop*[Title/Abstract]) OR (Colonoscopic Surgical Procedure*[Title/Abstract])) OR (Colonoscopic Surger*[Title/Abstract])) OR (Surgical Endoscop*[Title/Abstract])) OR (Endoscopic Surgical Procedure*[Title/Abstract])) OR (endoscop*[Title/Abstract]) |
| 4 | ("Colonoscopy"[Mesh]) OR "Endoscopy"[Mesh] |
| 3 | #1 OR #2 |
| 2 | (((((((((((((Artificial Intelligence*[Title/Abstract]) OR (Computational Intelligence*[Title/Abstract])) OR (Machine Intelligence*[Title/Abstract])) OR (Computer Reasoning[Title/Abstract])) OR (Computer Vision System*[Title/Abstract])) OR (AI[Title/Abstract])) OR (convolutional neural network*[Title/Abstract])) OR (Computer Neural Network*[Title/Abstract])) OR (Neural Network Model*[Title/Abstract])) OR (Neural Network*[Title/Abstract])) OR (Deep Learning[Title/Abstract])) OR (Hierarchical Learning[Title/Abstract])) OR (Machine Learning[Title/Abstract])) OR (Transfer Learning[Title/Abstract]) |
| 1 | ((("Artificial Intelligence"[Mesh]) OR "Neural Networks, Computer"[Mesh]) OR "Deep Learning"[Mesh]) OR "Machine Learning"[Mesh] |

| **Embase search strategy** | |
| --- | --- |
| No. | Query |
| #11. | #10 AND [embase]/lim NOT ([embase]/lim AND [medline]/lim) |
| #10. | #3 AND #6 AND #9 |
| #9. | #7 OR #8 |
| #8. | randomized controlled trial (topic)'/exp OR 'equivalence trial'/exp OR 'randomization'/exp OR 'double blind procedure'/exp OR 'single blind procedure'/exp OR 'placebo'/exp OR 'phase 3 clinical trial'/exp OR 'pragmatic trial'/exp OR 'controlled clinical trial'/exp OR 'controlled clinical trial (topic)'/exp |
| #7. | randomized controlled trial':ab,ti AND topic:ab,ti OR 'randomized controlled trial topic':ab,ti OR ('controlled clinical trial':ab,ti AND topic:ab,ti) OR 'controlled clinical trial topic'/exp OR 'controlled clinical trial topic' OR 'randomization'/exp OR randomization OR 'random allocation'/exp OR 'random allocation' OR (random AND allocation) OR 'double-blind method'/exp OR 'double-blind method' OR ('double blind' AND ('method'/exp OR method)) OR 'double blind procedure'/exp OR 'double blind procedure' OR (double AND ('blind'/exp OR blind) AND ('procedure'/exp OR procedure)) OR 'double-blind studies'/exp OR 'double-blind studies' OR ('double blind' AND ('studies'/exp OR studies)) OR 'single-blind method'/exp OR 'single-blind method' OR ('single blind' AND ('method'/exp OR method)) OR 'single blind procedure'/exp OR 'single blind procedure' OR (single AND ('blind'/exp OR blind) AND ('procedure'/exp OR procedure)) OR 'single-blind studies'/exp OR 'single-blind studies' OR ('single blind' AND ('studies'/exp OR studies)) OR (('control'/exp OR control) AND group*) OR random* OR sham OR placebo* OR ((singl* OR doubl*) AND adj AND (blind* OR dumm* OR mask*)) OR ((tripl* OR trebl*) AND adj AND (blind* OR dumm* OR mask*)) OR (control* AND adj3 AND ('study'/exp OR study OR 'studies'/exp OR studies OR trial* OR group*)) OR ((nonrandom* OR non) AND random*) OR 'non random*' OR 'quasi random*' OR quasirandom* OR allocated OR ((open AND label OR 'open label') AND adj5 AND ('study'/exp OR study OR 'studies'/exp OR studies OR trial*)) OR ((equivalence OR superiority OR 'non inferiority' OR noninferiority) AND adj3 AND ('study'/exp OR study OR 'studies'/exp OR studies OR trial*)) OR 'pragmatic study' OR (pragmatic AND ('study'/exp OR study)) OR 'pragmatic studies' OR (pragmatic AND ('studies'/exp OR studies)) OR ((pragmatic OR practical) AND adj3 AND trial*) OR ((quasiexperimental OR 'quasi experimental') AND adj3 AND ('study'/exp OR study OR 'studies'/exp OR studies OR trial*)) OR 'phase adj3 adj3' OR (phase AND (iii OR '3') AND adj3 AND ('study'/exp OR study OR 'studies'/exp OR studies OR trial*)) |
| #6. | #4 OR #5 |
| #5. | colonoscopy'/exp OR 'endoscopy'/exp |
| #4. | colonoscop*:ti,ab,kw OR 'colonoscopic surgical procedure*':ti,ab,kw OR 'colonoscopic surger*':ti,ab,kw OR 'surgical endoscop*':ti,ab,kw OR 'endoscopic surgical procedure*':ti,ab,kw OR endoscop*:ti,ab,kw |
| #3. | #1 OR #2 |
| #2. | artificial intelligence'/exp OR 'artificial neural network'/exp OR 'machine learning'/exp |
| #1. | artificial intelligence*':ti,ab,kw OR 'computational intelligence*':ti,ab,kw OR 'machine intelligence*':ti,ab,kw OR 'computer reasoning':ti,ab,kw OR 'computer vision system*':ti,ab,kw OR ai:ti,ab,kw OR 'convolutional neural network':ti,ab,kw OR 'computer neural network*':ti,ab,kw OR 'neural network model*':ti,ab,kw OR 'neural network*':ti,ab,kw OR 'deep learning':ti,ab,kw OR 'hierarchical learning':ti,ab,kw OR 'machine learning':ti,ab,kw OR 'transfer learning':ti,ab,kw |

| **Cochrane library search strategy** | |
| --- | --- |
| No. | Query |
| #1 | (Artificial Intelligence*):ti,ab,kw OR (Computational Intelligence*):ti,ab,kw OR (Machine Intelligence*):ti,ab,kw OR (Computer Reasoning):ti,ab,kw OR (Computer Vision System*):ti,ab,kw (Word variations have been searched) |
| #2 | (AI):ti,ab,kw OR (convolutional neural network*):ti,ab,kw OR (Computer Neural Network*):ti,ab,kw OR (Neural Network Model*):ti,ab,kw OR (Neural Network*):ti,ab,kw |
| #3 | (Deep Learning):ti,ab,kw OR (Hierarchical Learning):ti,ab,kw OR (Machine Learning):ti,ab,kw OR (Transfer Learning):ti,ab,kw |
| #4 | #1 OR #2 OR #3 |
| #5 | MeSH descriptor: [Artificial Intelligence] explode all trees |
| #6 | MeSH descriptor: [Neural Networks, Computer] explode all trees |
| #7 | MeSH descriptor: [Deep Learning] explode all trees |
| #8 | MeSH descriptor: [Machine Learning] explode all trees |
| #9 | #4 OR #5 OR #6 OR #7 OR #8 |
| #10 | (Colonoscop*):ti,ab,kw OR (Colonoscopic Surgical Procedure*):ti,ab,kw OR (Colonoscopic Surger*):ti,ab,kw OR (Surgical Endoscop*):ti,ab,kw OR (Endoscopic Surgical Procedure*):ti,ab,kw |
| #11 | (endoscop*):ti,ab,kw |
| #12 | #10 OR #11 |
| #13 | MeSH descriptor: [Colonoscopy] explode all trees |
| #14 | MeSH descriptor: [Endoscopy] explode all trees |
| #15 | #12 OR #13 OR #14 |
| #16 | (random*):ti,ab,kw OR (placebo*):ti,ab,kw |
| #17 | (singl*):ti,ab,kw OR (doubl*):ti,ab,kw OR (trebl*):ti,ab,kw OR (trip*):ti,ab,kw |
| #18 | (mask*):ti,ab,kw OR (blind*):ti,ab,kw OR (dumm*):ti,ab,kw |
| #19 | #17 AND #18 |
| #20 | #16 OR #19 |
| #21 | MeSH descriptor: [Randomized Controlled Trial] explode all trees |
| #22 | MeSH descriptor: [Randomized Controlled Trials as Topic] explode all trees |
| #23 | MeSH descriptor: [Random Allocation] explode all trees |
| #24 | MeSH descriptor: [Double-Blind Method] explode all trees |
| #25 | MeSH descriptor: [Single-Blind Method] explode all trees |
| #26 | MeSH descriptor: [Placebos] explode all trees |
| #27 | #20 OR #21 OR #22 OR #23 OR #24 OR #25 OR #26 |
| #28 | #9 AND #15 AND #27 |
